# Relationship between Community Collectivization and Financial Vulnerability of Female Sex Workers in Southern India

**DOI:** 10.1371/journal.pone.0156060

**Published:** 2016-05-26

**Authors:** Sangram Kishor Patel, Parimi Prabhakar, Anrudh Kumar Jain, Niranjan Saggurti, Rajatashuvra Adhikary

**Affiliations:** 1 HIV and AIDS Program, Population Council, 142, Golf Links, New Delhi, India; 2 India HIV/AIDS Alliance, Sarovar Center, Secretariat Road, Hyderabad, India; 3 Population Council, New York, United States of America; 4 Bill and Melinda Gates Foundation, New Delhi, India; Simon Fraser University, CANADA

## Abstract

**Introduction:**

Studies exploring the linkages between financial vulnerabilities and community collectivization of female sex workers (FSWs) are scarce in India despite having potential policy implications. To fill this gap in the literature, this study attempts to understand the financial vulnerabilities among FSWs and assess the relationship between community collectivization and financial vulnerabilities in southern India.

**Data and Methods:**

Data were drawn from a cross-sectional, behavioral tracking survey (BTS)—2014, conducted among FSWs (N = 2400) in Andhra Pradesh, a southern state of India under the Avahan-India AIDS initiative program. Adjusted odds ratios (AOR) and their 95% confidence intervals (CI) were estimated through multivariate logistic regression, to assess the independent relationships of the degree of community collectivization indicators with financial vulnerability indicators, adjusting for socio-demographic characteristics.

**Results:**

Most FSWs (87%) reported having either one or more financial vulnerability and nearly one-fifth had a high financial vulnerability. The risk of facing financial vulnerability was significantly lower among FSWs with a high degree of perceived collective efficacy (15% vs 31%; AOR: 0.4; 95% CI: 0.3–0.5) and collective agency (4% vs 21%; AOR: 0.2; 95% CI: 0.1–0.3) as compared to their respective counterparts, after controlling for their individual socio-demographic characteristics. FSWs with a high degree of collective efficacy are also less likely to report different components of financial vulnerability (e.g. income, saving, expenditure, and debt).

**Conclusion:**

This study finding suggests that community-led interventions such as improving collectivization are promising strategies to address financial vulnerabilities and a path to a sustainable reduction of HIV risk. This study calls for further evidence-based research and measurement of the effects of community-led approaches in addressing the financial vulnerabilities of the key population at risk for HIV.

## Introduction

Over the years, it has been recognized that HIV prevention requires structural interventions to address the vulnerabilities of sex workers, including legal, physical, social and economic factors [1, 2]. UNAIDS guidelines emphasize that human rights-based approaches are the standard for HIV prevention interventions, noting that the most successful interventions occur when “female sex workers (FSWs) are able to assert control over their working environments, negotiate and insist on safer sex” [3]. Community mobilization is an intervention strategy that encourages collectivization to bring about structural change [1]. Community mobilization aims not only to empower marginalized key populations (particularly FSWs) as a group for vulnerability reduction, but also increasingly allows them to make decisions and shape their own lives, which in turn influences the adoption and maintenance of low-risk behaviors [4, 5, 6]. Community-led interventions seek to change social and political structures by organizing FSWs to confront structural barriers at multiple levels, resulting in both individual and collective empowerment [7]. The Sonagachi Project, a community mobilization project with a secondary impact on economic strengthening, is one of the best-known FSW interventions, which is also included as an example of the best practice and a designated HIV-prevention model of the World Health Organization [8]. Inspired by the Sonagachi Project, Avahan- India AIDS Initiative program in India is also known for its combination approach to HIV prevention, including facilitating structural change through community mobilization. Avahan program launched an HIV prevention intervention in 2003 with key populations across six high-HIV prevalence states in India with one of the goals was to mobilize communities of FSWs and high-risk men who have sex with men/ Transgenders (HR-MSM) to manage and implement prevention programs themselves [9]. However, though microfinance appears as an intervention strategy in some Avahan programs [7], the economic aspect of the program has received very little attention and has not been properly explored in India. Previous studies have shown that high degree of community collectivization has better HIV knowledge [10], consistent condom use [11, 12, 13, 14, 15] and health service utilization [14, 16, 17], and lessened prevalence of sexually transmitted infections (STIs) [12, 18] among key populations; however its relationship with the financial vulnerability of sex workers has not been fully investigated in India. For researchers, programmers and policy-makers interested in developing economic and empowerment programs for people engaged in sex work, an understanding of their financial situation, accessibility of financial services, and challenges will be crucial in ensuring that such programs target the priorities and needs of this vulnerable population. At the same time, it will be useful to know, how community mobilization activities enable sex workers to reduce their financial vulnerability. Studies exploring community collectivization of FSWs and its relationship with financial vulnerability are scarce in India, despite having potential policy implications. This study attempts to fill this research gap by understanding the financial vulnerabilities of FSWs and assessing its association with community collectivization in southern India.

## Data and Methods

### Study settings and design

We used data from the Behavioral Tracking Survey (BTS)—2014 for this study. BTS is a cross-sectional survey conducted among FSWs who were members of any community-based organizations (CBOs) during April—May 2014 in Andhra Pradesh, a southern state of India. Its objective was to monitor the HIV prevention activities of the Avahan program. The survey collected information on community mobilization, STI treatment, HIV testing, condom use, social entitlements and financial inclusion among FSWs.

As part of the community mobilization initiative under the Avahan program, the India HIV/AIDS Alliance has been actively strengthening CBO activities in Andhra Pradesh. Avahan’s Common Minimum Program (CMP) for community mobilization among populations at risk of HIV infection (FSWs and HR-MSM) was introduced in mid-2012 to focus on:- organizational development—governance, leadership, project management; resource mobilization, financial inclusion, savings and loans, advocacy for HIV services, access to social entitlements and crisis response systems [19]. This program work closely with key populations to help them build CBOs that can advocate for the basic needs of their members through community-based groups (CBGs). CBGs under different CBOs, are empowered and have an understanding about shared responsibility towards increased access and utilization of project services (includes group savings at CBG level). Keeping sustainability as a major focus, the India HIV/AIDS Alliance has taken a strategic decision to organize the community into CBGs to sustain the CBOs. In each community mobilization implemented district, CBOs at the block levels are further divided into two organizations: (i) Site Level Associations (SLAs—at the mandal/cluster/ward level) and (ii) CBGs as grassroots/primary organizations. Usually, one CBG has approximately 10–15 registered FSWs. Each SLA is monitored by a CBG facilitator (one CBG facilitator for approximately 200–250 registered FSWs). One CBO has been formed for approximately 2000–3000 registered FSWs [19].

### Sampling and sample size

In the BTS-2014, six districts (Ananthapur, Chittor, Karimnagar, Khammam, Nalgonda and Warangal) were selected considering the geographical location and socio-cultural variability across the eight districts where the community collectivization activities were being implemented as part of the Avahan program through community-based organizations (CBOs). In each of the selected district, a sample of 400 FSWs was interviewed in the survey. The sample size at district-level was calculated based on a number of assumptions, including the prevalence of consistent condom use (CCU), expected change in CCU over the next few years, confidence level, the power of the estimate, design effect, etc. The sampling frame was prepared by taking the number of FSWs registered in each CBO. A two-stage cluster random sampling method was used to select respondents within the CBOs. In the first stage, the required numbers of CBGs within different clusters/wards were selected based on probability proportional to size. In the second stage, the required numbers of FSWs were randomly selected for the interview within each selected CBG. A total of 2400 FSWs were interviewed across all selected districts. All interviews were conducted by trained female investigators with verbal and written skills in Telugu, the local language of Andhra Pradesh. The survey instrument (structured questionnaire for FSWs) was developed in English and translated into Telugu. The questionnaire was pre-tested in communities similar to the survey sites, prior to the main survey. All the interviews were held in a private location specifically hired for the survey, or in a location convenient to the study participants. Field staff checked the data immediately after the interviews to ensure accuracy and completeness of the filled-in questionnaires. A user-written computer program in CSPro (version 5.0) was used for double data entry by trained data entry officers.

### Ethics statement

The overall BTS design, questionnaires and consent processes were reviewed and approved by the institutional review boards of Family Health International and the Karnataka Health Promotion Trust. Verbal consent was obtained from all respondents prior to participation in the interview, and steps were taken to ensure their confidentiality. On receipt of verbal consent from the eligible respondent, the interviewer signed the oral consent form on behalf of the respondent. In this survey, oral consent was recommended as information on the respondent’s background and behavioral characteristics were collected and no blood collection was done. For this survey, females aged 18 years or more who had sex in exchange of cash/kind in the last one month were recruited as FSWs and the information collected accordingly. No names and addresses were recorded on the questionnaires. Participants were not provided any compensation for their time in the study but were referred to local project services run by implementing agencies in the study districts.

### Measures

The socio-demographic variables considered in the analysis were age (<30 years, > = 30 years); marital status (never married, currently married, and widowed/divorced/separated/deserted); formal education (no, yes); usual place of practicing sex work (rural, urban/ semi-urban); and place of solicitation or typology for sex work (home, public places/street, brothel/lodge, mobile phones).

Globally, community mobilization has been defined and operationalized in numerous ways and includes concepts from a range of approaches, including participatory development [20], social policy [21] and structural interventions [22]. Avahan program in India describes community mobilization as the procedure by which key population members ‘‘utilize their intimate knowledge of vulnerability to overcome the barriers they face and realize reduced HIV risk and greater self-reliance through their collective action” [9]. This study adopts the community mobilization concepts from the Avahan program [9, 22]. Three independent variables comprising community mobilization indicators were considered in the analysis: collective efficacy, collective agency and collective action; each of these variables were composite indices based on a series of items.

Collective efficacy refers to FSWs’ belief in the power of the community to work together to bring about positive change [9, 22, 23, 24, 25, 26]. This was measured based on responses to the question: How confident are you that FSWs in your community can work together to achieve the following goals: keep each other safe from harm; increase condom use with clients; speak up for your rights; and improve your lives? Responses to these questions included: 1 = not at all, 2 = somewhat, 3 = very and 4 = completely confident. Using these four questions and corresponding responses, an index was constructed, which had a reliability (Cronbach’s alpha) of 0.82. The index scores were further divided into two equal categories based on the mid-value of collective efficacy: 0 = low (1–2.4999) and 1 = high (2.5–4).

Collective agency refers to the choice, control and power that FSWs have to act for themselves to claim their rights (whether civil, political, economic, social or cultural) and to hold others accountable for these rights [9, 22, 23, 24, 25, 26]. This indicator was measured based on responses to the question: In the past 6 months, have you negotiated with or stood up against the following stakeholders- police, madam/broker, local goon (gang member), clients or any other sexual partner- in order to help a fellow sex worker or to help fellow sex workers? A separate question for each of the above stakeholders was asked, with the possible binary response categories ‘Yes’ (coded as 1) and ‘No’ (coded as 0). Using these four questions and corresponding responses, an index was constructed, which had a reliability (Cronbach’s alpha) of 0.60. The index scores were further divided into two equal categories based on the mid-value of collective agency: 0 = low (0–0.4999) and 1 = high (0.5–1).

Collective action refers to the strategic and organized activities of mobilized community members to increase the community’s visibility and present or enact its agenda for change (for example, through rallies, demonstrations, or meetings with stakeholders) [9, 22, 23, 24, 25, 26]. This was measured based on responses to the following seven questions: Whether the sex workers group comes together to demand/help for the following: (1) ration card, (2) voters card, (3) bank account, (4) free education for children, (5) health insurance, (6) representation in government forums, and (7) better health services from the government. A separate question was asked for each of the above social entitlements and services, with the possible binary response categories ‘Yes’ (coded as 1) and ‘No’ (coded as 0). Using these seven questions and corresponding responses, an index was constructed, which had a reliability (Cronbach’s alpha) of 0.83. The index scores were further divided into two equal categories based on the mid-value of collective action: 0 = low (0–0.4999) and 1 = high (0.5–1).

### Financial Vulnerability

Financial vulnerability depends on various factors, both outside a person's control, such as adverse economic conditions, and those that are specific, including levels of savings or debt. As per Fin Mark Trust (2009), a financial vulnerability index must reflect the overall vulnerability of a person and should also capture the different dynamics influencing a person’s profile for income, savings, expenditure and debt [27]. A similar construct has been used in this study where the financial vulnerability index is defined on the basis of the combined scores of income vulnerability, saving vulnerability, expenditure vulnerability and debt vulnerability of FSWs. FSWs’ income vulnerability is defined as those who do not have any occupation other than sex work, or currently not receiving social schemes/grants from government and other sources, or not able to access/transfer money from family and friends (No = 0, Yes = 1); FSWs’ saving vulnerability is defined as those who do not have money for future or emergencies expecting when income will be less, or do not have any personal or family assets (own house, agriculture land and other resources) (No = 0, Yes = 1); FSWs’ expenditure vulnerability is those who do not have money of their own and do not have independency to make expenditure of their own (No = 0, Yes = 1); and FSWs’ debt vulnerability is defined as those who were under financial debt at the time of survey (No = 0, Yes = 1). The combined scores were calculated to measure the overall financial vulnerability of FSWs (e.g. 0 = no financial vulnerability, 1 = any one financial vulnerability, 2 = any two, 3 = any three and 4 = having all four types of financial vulnerability). For the purpose of the multivariate analysis, the scores were further grouped into two categories (0 = low/medium vulnerability (< 3 score) and 1 = high vulnerability (> = 3 score).

### Data Analysis

Descriptive statistics (i.e., means, standard deviations, and proportions) and bivariate analyses were used to describe the strength and association of socio-demographic characteristics, community mobilization of FSWs and the financial vulnerability indicators. The respective p-values for the bivariate analysis were calculated through chi-square test. Adjusted odds ratios (AOR) and their 95% confidence intervals (CI) were estimated through multivariate logistic regression, to assess the independent relationships of degree of community collectivization indicators with financial vulnerability indicators, adjusting for socio-demographic characteristics. All analyzes described above were conducted using STATA software (version 11.2).

## Results

**Fig 1:**
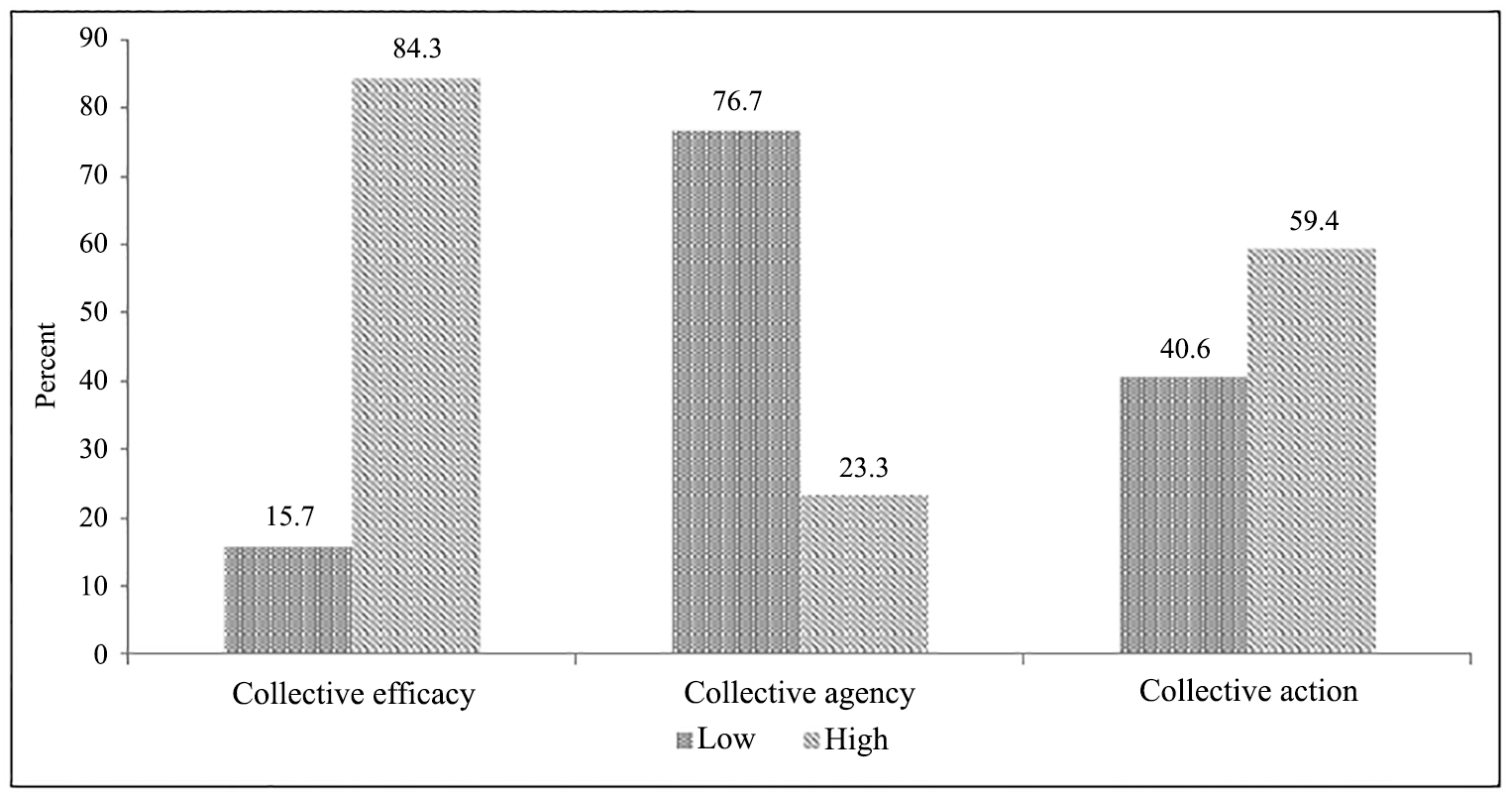
Distribution of levels of community collectivization indicators among female sex workers (N = 2400) in Andhra Pradesh, India, 2014. **Note:** The collective efficacy index was divided into two equal categories based on the mid-value as: 0 = low and 1 = high; The collective agency index was divided into two equal categories based on the mid-value as: 0 = low and 1 = high; The collective action index was divided into two equal categories based on the mid-value as: 0 = low and 1 = high.

Most FSWs (87%) reported having either one or more forms of financial vulnerabilities and nearly one-fifth (17%) had a high financial vulnerability (Table 1). Two-thirds reported having the debt vulnerability (66%), a little more than half had the expenditure vulnerability (55%), one-fourth had the savings vulnerability (28%) and almost one in ten had the income vulnerability (8%). Overall, financial vulnerability (high) was significantly higher among FSWs who were never married (18%), or widowed/deserted/separated/divorced (22%), those practicing sex work in urban areas (22%), those using the street or public place (20%) and the mobile phone (18%) as their main mode of sex solicitation (Table 2). More than eight out of ten (84%) sex workers reported a high degree of collective efficacy, nearly three-fifths reported a high degree of collective action (59%) and 23% reported a high degree of collective agency (Fig 1).

**Table 1:** Financial vulnerability as reported by female sex workers (N = 2400) in Andhra Pradesh, India, 2014.

Indicators	Percentage	N
*Income vulnerability*:		
Having activities/occupation other than sex work, or currently receiving social schemes/grants from govt. & other sources, or able to access transfer money from family and friends		
No	7.6	183
Yes	92.4	2217
*Saving vulnerability*:		
Saved money for future or emergencies expect when income will be less, or having personal/family assets (own house, agriculture land and other resources)		
No	28.0	672
Yes	72.0	1728
*Expenditure vulnerability*:		
Having money of their own and decide how to make expenditure		
No	55.0	1321
Yes	45.0	1079
*Debt vulnerability*:		
Not under financial debt at the time of survey		
No	66.0	1586
Yes	34.0	814
Financial vulnerability scores		
0	12.6	302
1	36.3	871
2	34.0	817
3	16.0	383
4	1.1	27
Financial vulnerability Index		
Low/Medium	82.9	1990
High	17.1	410
**Total**	**100.0**	**2400**

**Note**: Financial vulnerability index was calculated based on the combine scores of income, saving, expenditure and debt vulnerabilities. The financial vulnerability score ranges from 0 to 4. The combined scores were divided into two categories (0 = low/medium (< 3 score) and 1 = high (> = 3 score).

**Table 2:** Socio-demographic profile and financial vulnerabilities among female sex workers (N = 2400) in Andhra Pradesh, India, 2014.

		Vulnerability						
Socio-demographic characteristics	% (n) or Mean (SD)	No other source of income rather than sex work	No saving for future and not having personal assets	Not having money of their own and decide how to make expenditure	Currently under financial debt	Financial vulnerability		
						Low/Medium	High	p-value
**Age (Mean, SD)**	30.9 (5.8)							
**Age**								0.884
<30 years	43.3 (1040)	9.8 (102)	29.5 (307)	56.0 (582)	63.8 (663)	82.8 (861)	17.2 (179)	
> = 30 years	56.7 (1360)	6.0 (81)	26.8 (365)	54.3 (739)	67.9 (923)	83.1 (1129)	16.9 (231)	
**Education**								0.116
No formal education	56.3 (1350)	6.7 (91)	29.1 (393)	56.0 (757)	67.9 (916)	81.8 (1105)	18.2 (245)	
Having formal education	43.7 (1050)	8.8 (92)	26.6 (279)	53.7 (564)	63.8 (670)	84.3 (885)	15.7 (165)	
**Marital status**								0.000
Never married	5.0 (119)	23.5 (28)	19.3 (23)	50.4 (60)	57.1 (68)	82.3 (98)	17.7 (21)	
Currently married	66.5 (1596)	6.6 (105)	26.1 (417)	54.4 (868)	66.4 (1059)	85.0 (1358)	15.0 (238)	
Widowed/deserted/separated/ divorced	28.5 (685)	7.3 (50)	33.9 (232)	57.4 (393)	67.0 (459)	78.0 (534)	22.0 (151)	
**Typology for sex work**								0.007
Home-based	13.9 (333)	3.9 (13)	23.7 (79)	49.3 (164)	64.3 (214)	88.0 (293)	12.0 (40)	
Brothel and lodge-based	4.5 (109)	2.8 (03)	13.8 (15)	47.7 (52)	78.0 (85)	89.0 (97)	11.0 (12)	
Street/public places	27.6 (663)	6.3 (42)	30.0 (199)	54.6 (362)	67.7 (449)	80.4 (533)	19.6 (130)	
Mobile phones	54.0 (1295)	9.7 (125)	29.3 (379)	57.4 (743)	64.7 (838)	82.4 (1067)	17.6 (228)	
**Usual place of practicing sex work**								0.000
Rural/semi-urban	46.7 (1121)	5.6 (63)	18.7 (210)	45.5 (510)	72.0 (806)	88.8 (996)	11.2 (125)	
Urban	53.3 (1279)	9.4 (120)	36.1 (462)	63.4 (811)	61.0 (780)	77.7 (994)	22.3 (285)	
**Total**	**100.0 (2400)**	**7.6 (183)**	**28.0 (672)**	**55.0 (1321)**	**66.0 (1586)**	**82.9 (1990)**	**17.1 (410)**	

Note: p-values for financial vulnerability were calculated through chi-square test.

Table 3 presents the results of the association between community mobilization indicators and financial vulnerability after controlling for FSWs’ age, education, marital status, typology of sex work, usual place of practicing sex work and reasons for entry into sex work. The results show that the likelihood of reporting income vulnerability was significantly lower among FSWs with high collective efficacy (5% vs 8%; AOR: 0.7; 95% CI: 0.4–0.9) compared to FSWs with low/medium vulnerability. The likelihood of reporting savings vulnerability was significantly lower among FSWs with a high degree of collective efficacy (26% vs 40%; AOR: 0.5; 95% CI: 0.4–0.6), collective agency (5% vs 35%; AOR: 0.2; 95% CI: 0.1–0.3) and collective action (23% vs 35%; AOR: 0.6; 95% CI: 0.5–0.7) as compared to their corresponding counterparts. The probability of reporting expenditure vulnerability was significantly lower among FSWs with a high degree of collective efficacy (54% vs 60%; AOR: 0.8; 95% CI: 0.6–0.9) and collective agency (41% vs 60%; AOR: 0.5; 95% CI: 0.4–0.6), whereas it was marginally higher among FSWs with high collective action (53% vs 57%; AOR: 1.3; 95% CI: 1.1–1.6) as compared to others. The odds of reporting debt vulnerability were significantly higher among FSWs with a high degree of collective agency (78% vs 63%; AOR: 1.9; 95% CI: 1.6–2.4) and collective action (70% vs 60%; AOR: 1.4; 95% CI: 1.2–1.7), whereas the chances of reporting debt vulnerability were 20% lesser among FSWs with high collective efficacy (65% vs 71%; AOR: 0.8; 95% CI: 0.6–0.9). The likelihood of overall financial vulnerability was significantly lower among FSWs with a high degree of collective efficacy (15% vs 31%; AOR: 0.4; 95% CI: 0.3–0.5) and collective agency (4% vs 21%; AOR: 0.2; 95% CI: 0.1–0.3).

**Table 3:** Association between community mobilization indicators and financial vulnerability among female sex workers (N = 2400) in Andhra Pradesh, India, 2014.

	Vulnerability									
Community mobilization indictors	No other source of income rather than sex work		No saving for future and not having personal assets		Not having money of their own and decide how to make expenditure		Currently under financial debt		Financial vulnerability (High)	
	%	AOR (95% CI)	%	AOR (95% CI)	%	AOR (95% CI)	%	AOR (95% CI)	%	AOR (95% CI)
Collective efficacy										
Low	8.1	Referent	40.0	Referent	59.8	Referent	71.3	Referent	30.6	Referent
High	5.0	0.7 (0.4–0.9)*	25.8	0.5 (0.4–0.6)***	54.0	0.8 (0.6–0.9)**	65.1	0.8 (0.6–0.9)**	14.6	0.4 (0.3–0.5)***
Collective agency										
Low	8.0	Referent	34.9	Referent	59.4	Referent	62.6	Referent	21.0	Referent
High	6.3	0.8 (0.5–1.1)	5.4	0.2 (0.1–0.3)***	40.6	0.5 (0.4–0.6)***	77.5	1.9 (1.6–2.4)***	4.3	0.2 (0.1–0.3)***
Collective action										
Low	8.4	Referent	35.4	Referent	53.0	Referent	60.3	Referent	19.4	Referent
High	7.1	0.9 (0.7–1.4)	23.0	0.6 (0.5–0.7)***	56.5	1.3 (1.1–1.6)	70.0	1.4 (1.2–1.7)***	15.5	0.8 (0.7–1.1)

Note: CI: Confidence Interval;

*, ** and *** indicate values are significant at 10%, 5% and 1% level of significance;

AOR: Adjusted Odds Ratios; AORs are adjusted for age, education, marital status, typology, usual place of practicing sex work.

## Discussion

The study findings indicate that most FSWs have either income, saving, expenditure or debt vulnerabilities, and nearly one-fifth have a high financial vulnerability in Andhra Pradesh, India. The high level of financial vulnerability is significantly more among FSWs who are never married or widowed/deserted/separated/divorced, those practicing sex works in urban areas, and those using the street or public places, and mobile phones, as the primary mode of sex solicitation. The findings of this study illustrate that the community collectivization indicators (mainly collective efficacy and collective agency) are significantly associated with financial vulnerability. The findings show that FSWs who report a high degree of collective efficacy are less likely to report financial vulnerability as well as different components of financial vulnerability (income, saving, expenditure, and debt). The results of this study add to the existing evidence, which suggests the association between FSWs’ high degree of community collectivization (more specifically, collective efficacy) and higher consistent condom use with various types of partners and STI treatment-seeking from government health facilities [13, 14, 15, 23, 28, 29]. This study adds further that a high degree of the collective efficacy has an inverse association with FSWs’ financial vulnerability. The study findings further highlight that FSWs with a high degree of collective agency are less likely to experience savings vulnerability, expenditure vulnerability and financial vulnerability, although are more likely to have debt vulnerability. FSWs with a high degree of collective action are significantly less likely to face saving vulnerability, but are more likely to report debt vulnerability. In the recently implemented Avahan-III program, most of the CBOs offer access to micro-savings and credit to FSWs with the aim of empowering them through reduced economic dependence on sex work and enhanced economic security. In some of the cases, FSWs are not able to access the services offered by formal financial institutions, either due to their inability to fulfill the required criteria or due to stigma and discrimination, which forces them to borrow from informal exploitative sources that charge exorbitant rates of interest. A recent study among FSWs in one of the southern states of India reported that membership in multiple microfinance groups has increased their credit burden subsequently leading to unpaid and unprotected sex work to repay the debt [24]. In most of the instances, they are also subject to harassment from madam, local goons, police and pimps who extort their money. FSWs are caught in a perpetual cycle of debt and with very little money for their daily sustenance and survival of their families. Findings from this study suggest that more than 66% of FSWs were in debt at the time of the survey in Andhra Pradesh and more than half of the sex workers have less independence to spend money of their own, and always need permission from someone else for their daily expenses. Considering these above points, it may be possible that despite having higher collectivization (particularly with the high level of collective agency and collective action), FSWs have increased debt vulnerability.

This study suggests that all the community mobilization indicators are consistent in explaining that FSWs with a high degree of collectivization are more likely to save for future emergencies. These findings are well supported by other studies, which show that high-risk populations, including FSWs with high collectivization are more likely to have individual bank accounts and save money in group saving schemes [25, 26, 30]. In addition, FSWs with high collectivization (particularly, collective efficacy and collective agency) are more likely to have access to social schemes or grants and at least one social entitlement [26, 30]. The findings of this study reveal that there is a need to develop strategies to improve community collectivization among FSWs, particularly collective agency and collective action; given that the collective agency has reduced over time as compared to the findings from the previous studies from same geographies [14, 15, 16]. This study further highlights a strong need for programs to address financial vulnerabilities and to reduce the debt vulnerability in particular. These interventions should be similar to the concept of “transformatory” interventions, where goals are set by beneficiaries as active agents, rather than “instrumental”, where goals are set outside [3, 31]. The best examples in India are the Sonangachi project in Kolkata, and the Pragati intervention in Bangalore, where community-led interventions helped change community perceptions of HIV/AIDS, condom use, STI prevention, and sex work, and reduce stigma, culminating in a political-legal change that helped FSWs organize and claim their rights. It can be underlined that mobilizing sex workers was the first step towards generating change in the community, and economic strengthening activities (e.g. microfinance and vocational training) followed, based on the response from the FSW community [8, 32]. The Karnataka Health Promotion Trust (KHPT), in one of the southern states in India, also provides an example of an HIV prevention program that emphasizes community mobilization for the economic strengthening of sex workers [24].

Although the study findings offer important insights on the relationship between community collectivization and financial vulnerability among FSWs in India, they must be interpreted in light of certain study limitations. The community mobilization and financial vulnerability indicators are based on self-reports which are susceptible to social desirability and recall biases. The analyzes are cross-sectional and causality cannot be assumed as in the case of prospective research studies. Further prospective research is needed to test if community-led interventions reduce FSWs’ financial vulnerabilities. The differences were seen in collective efficacy, collective agency, and collective action may also be caused by varying degrees of participation in the intervention activities, innate differences in characteristics (such as personality and personal experiences), that may drive differences in the constructs or some unmeasured confounder as well. Furthermore, the study findings cannot be generalized to the whole FSWs community as the study was conducted among FSWs who were members of CBOs at the time of the survey. In conclusion, the study findings offer important insights into furthering community-led interventions for addressing the financial vulnerability, as a path to a sustainable reduction of HIV risk. The study also recommends that more research is needed on savings-led microfinance and combined microfinance/vocational training interventions, and their association with community collectivization. More importantly, HIV prevention programs and policies need to acknowledge that financial vulnerability is an important structural issue that needs to be addressed as part of community collectivization interventions. More in-depth research is required to investigate the causal mechanisms of effective economic strengthening interventions, including their effect on poverty, debt and food insecurity reductions, which have implications for ending the spread of HIV in India and elsewhere.
